# MPI Phantom Study with A High-Performing Multicore Tracer Made by Coprecipitation

**DOI:** 10.3390/nano9101466

**Published:** 2019-10-16

**Authors:** Harald Kratz, Azadeh Mohtashamdolatshahi, Dietmar Eberbeck, Olaf Kosch, Ralf Hauptmann, Frank Wiekhorst, Matthias Taupitz, Bernd Hamm, Jörg Schnorr

**Affiliations:** 1 Charité-Universitätsmedizin Berlin, corporate member of Freie Universität Berlin, Humboldt-Universität zu Berlin, and Berlin Institute of Health, Department of Radiology, D-10117 Berlin, Germany; Azadeh.Mohtashamdolatshahi@charite.de (A.M.); Ralf.Hauptmann@charite.de (R.H.); Matthias.Taupitz@charite.de (M.T.); Bernd.Hamm@charite.de (B.H.); Joerg.Schnorr@charite.de (J.S.); 2 Physikalisch-Technische Bundesanstalt, D-10587 Berlin, Germany; Dietmar.Eberbeck@ptb.de (D.E.); Olaf.Kosch@ptb.de (O.K.); Frank.Wiekhorst@ptb.de (F.W.)

**Keywords:** magnetic particle imaging (MPI), magnetic particle spectroscopy (MPS), magnetic nanoparticles (MNP), magnetic multicore particles (MCP), coprecipitation

## Abstract

Magnetic particle imaging (MPI) is a new imaging technique that detects the spatial distribution of magnetic nanoparticles (MNP) with the option of high temporal resolution. MPI relies on particular MNP as tracers with tailored characteristics for improvement of sensitivity and image resolution. For this reason, we developed optimized multicore particles (MCP 3) made by coprecipitation via synthesis of green rust and subsequent oxidation to iron oxide cores consisting of a magnetite/maghemite mixed phase. MCP 3 shows high saturation magnetization close to that of bulk maghemite and provides excellent magnetic particle spectroscopy properties which are superior to Resovist^®^ and any other up to now published MPI tracers made by coprecipitation. To evaluate the MPI characteristics of MCP 3 two kinds of tube phantoms were prepared and investigated to assess sensitivity, spatial resolution, artifact severity, and selectivity. Resovist^®^ was used as standard of comparison. For image reconstruction, the regularization factor was optimized, and the resulting images were investigated in terms of quantifying of volumes and iron content. Our results demonstrate the superiority of MCP 3 over Resovist^®^ for all investigated MPI characteristics and suggest that MCP 3 is promising for future experimental in vivo studies.

## 1. Introduction

Magnetic particle imaging (MPI) was first presented as a novel radiation-free imaging modality by Weizenecker and Gleich in 2005 [1]. MPI is capable of 4D imaging with high temporal resolution (46 volumes per second) and a spatial resolution of 1–2 mm depending on the scanner type and the tracer used. MPI directly and specifically measures the magnetic moments of magnetic nanoparticles (MNP) using an alternating magnetic field. Its high sensitivity and high temporal resolution with the option of quantifying the administered MNP as tracers make MPI a promising method, especially for imaging the cardiovascular system and local perfusion, if the tracer behaves as a blood pool agent [2,3]. The MNP have nonlinear magnetization behavior, generating higher harmonics of the applied excitation frequency which are measured inductively. Additional magnetic field gradients are used to facilitate spatial encoding in the field of view (FOV) [4]. For more detailed information on principles of MPI one can refer to [1]. As MPI only detects the tracer, combination of MPI with other imaging modalities such as magnetic resonance imaging (MRI) or computed tomography (CT) is necessary to obtain the corresponding anatomical information [5,6]. Both, MPI scanners and tracers are still under development. Regarding tracers, Resovist^®^ is still a kind of standard for MPI, since most published studies of MPI, especially those obtained by in vivo experiments, have been conducted with this tracer [2,7,8,9,10,11,12,13,14,15,16,17]. Since Resovist^®^ was initially developed as a contrast agent for MRI of the liver it has some disadvantages when used for MPI. For MPI investigations of the cardiovascular system the in vivo blood circulation time of Resovist^®^ is limited, and MPI signal intensity is also generally restricted [16]. Several other MNP with promising properties as MPI tracers have been described in the literature [16,18,19,20], but most of these MNP are currently not generally available for MPI studies. Furthermore, some potential MPI tracers with superior magnetic particle spectroscopy (MPS) signal intensities compared with Resovist^®^ are commercially available (perimag^®^ [21,22], synomag-D^®^ [23], micromod Partikeltechnologie GmbH, Rostock, Germany). Nevertheless, to further advance MPI as a medical imaging modality, there is still a great need for the development of new MPI tracers. Tracers with excellent MPI characteristics are especially crucial for the potential preclinical development of this imaging modality and the identification of new possible clinical applications. The most important methods for synthesizing new MNP are coprecipitation and thermal decomposition [5,24,25]. Both methods have their strengths and weaknesses. Thermal decomposition provides very good control over the MNP shape, and the achievable size distribution is very narrow. However, this method is complicated and needs high temperatures in some cases over 300 °C plus an inert gas atmosphere and produces a lot of possibly toxic byproducts [26]. Moreover, with this method, the MNP are synthesized in organic solvents and, for use in biological systems, need to be transferred into the aqueous phase. 

In contrast, coprecipitation is a very simple method that needs ambient conditions with a temperature of up to approx. 95 °C but often lacks good control over shape, and the realizable size distribution is only relatively narrow [24,27]. Apart from MPI tracers consisting of single-core particles, there are also some which are based on so-called multicore particles (MCP), which theoretically might have some advantages and are also partly contained in Resovist^®^ [28]. In theory, the ideal MPI tracer should have a magnetic core diameter of approx. 25 nm for a 25-kHz excitation field frequency and be monodisperse [29]. In addition, for in vivo use MPI tracers have to be biocompatible and biodegradable [5]. We already reported the synthesis of MCP [26], which is a new generation of MPI tracers with very good MPI tracer properties. In the first in vivo studies in rats biocompatibility of MCP was investigated with doses up to 3 mmol Fe/kg of body weight and no adverse side effects could be observed. In addition, MCP showed a fast MRI degradation in liver with a half-life of seven days [26]. Compared to the commercially available potential MPI tracers from micromod Partikeltechnologie GmbH our MCP have a different coating and magnetic core structure. Here we present the synthesis and physicochemical characterization of a further improved version of these MCP made by coprecipitation and investigations on their MPI signal generating properties in phantom experiments performed using a preclinical MPI scanner. We examined the new tracer focusing on the achievable sensitivity, spatial resolution, and occurrence and severity of artifacts using Resovist^®^ as standard of reference. In addition, we assessed tracer selectivity concerning the discrimination of small tracer volumes in close vicinity to each other, in particular with a view to future in vivo experiments and further advancement of MCP.

## 2. Materials and Methods 

### 2.1. Chemicals

Acrylamide solution (30% in water), N-N´-methylenebisacrylamide solution (2% in water), ammonium persulfate (1% in water), and N,N,N´,N´-tetramethylethylenediamine (≥98%, p.a.) were purchased from Carl Roth GmbH (Karlsruhe, Germany). All other chemicals were purchased from Sigma-Aldrich (Steinheim, Germany). Iron(II) chloride tetrahydrate and carboxymethyl dextran sodium salt were used as received without further purification. To prepare a 5% hydrogen peroxide solution (5 wt% in H_2_O_2_), hydrogen peroxide solution (30 wt% in H_2_O_2_) was diluted with five parts of deionized water. Deionized water was generally produced using a Mill-Q A10 system (Millipore, Billerica, MA, USA) and used for both synthesis and for preparation of solutions and dispersions. 

### 2.2. Magnetic Particle Imaging (MPI) Tracers

Resovist^®^ was purchased from Fujifilm RI Pharma Co., Tokyo, Japan. Optimized multicore particles (MCP 3) were synthesized in our laboratory at Charité using a technique of alkaline coprecipitation of ferrous chloride in the presence of atmospheric oxygen and subsequent oxidation by hydrogen peroxide [26]. Thereafter, the washed MNP were coated with carboxymethyl dextran sodium salt (CMD, 10–20 kD) and heated for several hours. After washing with Milli-Q water by ultrafiltration using Vivaflow 200 filters with a 100 kDa regenerated cellulose (RC) membrane (Sartorius AG, Göttingen, Germany), the resulting MNP were divided into different fractions by repeated magnetic separation (see supplement text S1 for further details). For later use, MCP were concentrated to 145.5 mmol Fe/l by centrifugation at 3112 × g using Amicon Ultra-15 Centrifugal Filter Units (PLHK Ultracel-PL Membrane, 100 kDa). Thereafter the dispersion was passed through 0.2 μm cellulose mixed ester (CME) syringe filters for sterile filtration. MNP dispersions were diluted with Milli-Q water to prepare the respective final concentrations required for the experiments and MNP characterization (see below). 

### 2.3. Magnetic Nanoparticles (MNP) Characterization

Hydrodynamic diameters of MNP were determined by dynamic light scattering (DLS) on a Zetasizer Nano ZS particle analyzer (Malvern Instruments, Worcestershire, UK). For DLS measurement, MNP dispersions were diluted with Milli-Q water to a final concentration of 1 mmol Fe/l. For ζ-potential measurement, MNP dispersions were diluted with 10 mM NaCl to a final concentration of 1 mmol Fe/l and adjusted to a pH of 7.19 with NaOH. MNP size and morphology were analyzed by transmission electron microscopy (TEM) using a TECNAI G2 20 S-Twin (FEI-Company, Hillsboro, OR, USA). Average core/multicore diameters (d_v_) and size distributions were calculated for each nanoparticle sample by averaging 200 MCP from the TEM images using ImageJ software (developed by the National Institutes of Health, Bethesda, Maryland, USA). The ferric and ferrous iron contents of the MNP dispersions were colorimetrically determined using the phenanthroline method [30]. MNP were also analyzed by magnetic particle spectroscopy (MPS) to assess the response of MNP to alternating magnetic fields. MPS measurements were performed using a magnetic particle spectrometer (MPS-3, Bruker BioSpin, Ettlingen, Germany) at 10 mT, 25 kHz, and 37 °C for 10 s. Deviating from that, samples embedded in polyacrylamide (PAA) gel were measured at 27.5 ± 1.5 °C. 

For measurements, 30 µL of each sample was filled in Life Technologies polymerase chain reaction (PCR) tubes. The amplitude of the magnetic moment was normalized to the iron content of each sample, resulting in the spectrum of the magnetization, *M_k_*, which is given in Am^2^/mol(Fe). For M(H) measurements, 75 µL sample volumes were filled in polycarbonate capsules. The magnetic moment of each sample was measured using an MPMS (Magnetic Property Measurement System, Quantum Design, USA) successively increasing the applied magnetic field from 0 to 5 T. The background signal caused by empty capsules, diamagnetic susceptibility of the dispersion medium, and deionized water was subtracted from the signal obtained for the samples. The resulting signal represents the magnetization of the MNP and was normalized to the iron content of the sample for quantitative evaluation. For partial immobilization of MCP 3 in PAA gel, 52 µL acrylamide solution (30% in water), 52 µL N-N´-methylenebisacrylamide solution (2% in water), 10 µL ammonium persulfate (1% in water), 273 µL water, 3 µL MCP 3 dispersion (145.5 mmol (Fe)/l), and 9.7 µL N,N,N´,N´-tetramethyletylenediamine (1:30 diluted with water (v/v)) were mixed and subsequently vortexed. Then 50 µL of the resulting dispersion was filled in a measuring cuvette and polymerized at 60 °C in a water bath for approx. 30 min. 

### 2.4. MPI Phantom Studies

The phantoms used in this study were manufactured from PVC tubes with 0.5 mm wall thickness and inner diameters of 1, 2, and 3 mm (Conrad Electronic, Hirschau, Germany). All tubes were cut to a final length of 20 mm. The tubes were filled with tracer dispersions and the open ends sealed with paraffin embedding wax (Paraplast, Tissue Embedding Medium, Sigma, St Louis, MO, USA). For the dual-tube phantoms, pairs of tubes with the same or different inner diameters were longitudinally fixed to each other using Parafilm M^®^ (Bemis Company, Neenah, Wi, USA). Single-tube phantoms of the dilution series were filled with 90 µL tracer dispersion with the following concentrations: 10, 5, 2, 1, 0.5, 0.2, and 0.1 mmol Fe/l. For the dual-tube phantom series, six different pairwise combinations of tubes were filled with MCP 3 or Resovist^®^ with in each case a concentration of 10 mmol Fe/l. In the third phantom series, tubes with the same inner diameters (1, 2, and 3 mm) were longitudinally fixed to each other and filled with 1 mmol Fe/l MCP 3 or Resovist^®^. 

The volumes of tracer dispersions were 90 µL for the PVC tubes with 3 mm inner diameter, 40 µL for the tubes with 2 mm inner diameter, and 10 µL for the tubes with 1 mm inner diameter. MPI phantom experiments were performed on a preclinical MPI scanner (Bruker 25/20 FF, Bruker Biospin GmbH, Ettlingen, Germany). The scanner operates according to the field-free-point (FFP) principle and needs a prerecorded system function (SF) for reconstruction of images. The standard 25/20 MPI system has dual-purpose coils to simultaneously generate the drive-field (DF) for excitation of the MNP dispersion and to receive the voltage signals from the MNP induced by magnetization. In addition, a prototype of a separate receive coil (developed by Bruker and Physikalisch-Technische Bundesanstalt (PTB)) was installed in the x-channel of the MPI system at Charité to improve the signal-to-noise ratio (SNR) and sensitivity [31,32]. In the MPI measurement, we applied a DF amplitude of 12 mT with approx. 25 kHz in all three directions and a selection field gradient of (Gx/Gy/Gz) = (1.25/1.25/2.5) T/m. The phantoms were measured in two different orientations to take the different gradient resolutions of x, y, and z direction into account.

### 2.5. Image Reconstruction and Analysis

#### 2.5.1. Reconstruction

Prior to reconstruction, the background signal was subtracted from the measured signal. To this end, the background signal was measured before the phantom measurement for the same length of time as required for the actual measurement [33]. The acquired data were reconstructed to 33 × 33 × 33 voxels using an iterative Kaczmarz algorithm [34] with five iteration steps (ParaVision 6, Bruker Biospin GmbH, Ettlingen, Germany). The voxel size of the applied SF was 0.8 × 0.8 × 0.4 mm^3^. For reconstruction, the frequency range above the SNR threshold, determined from the SF, was employed. For elimination of background noise, the bandwidth was limited to 0.09–125 MHz [33]. For both, MCP 3 and Resovist^®^, 2438 frequencies were chosen according to the SNR of the applied system function (SF), while different SNR thresholds of 24 and 6 were selected for MCP 3 and Resovist^®^, respectively. For SF parameters please see supplement text S6. The maximum order of mixing frequencies was 25 for all images. A block average of 5 was applied to each measurement in the reconstruction step to reduce image noise. In reconstruction of the phantom measurements, the determined SNR-optimized regularization factors λ between 10^−2^ and 10^−1^ were used for MCP 3 and Resovist^®^. In the final reconstruction of the phantoms, λ = 10^−2^ was used for higher concentrations, i.e., 10 and 5 mmol Fe/l, and λ = 10^−1^ for lower concentrations because these values were observed to be the optimal compromise for the two tracers.

#### 2.5.2. Evaluation of SNR-Optimized Regularization Factor (Plotting SNR Against λ)

SNR of different tracer concentrations in the dilution series was analyzed using MATLAB (Mathworks, Natick, MA, USA) in relation to different regularization factors (λ), ranging from 10^0^ to 10^−6^ [16]. To this end, images of the single-tube phantoms were reconstructed with 5 iterations. To determine the SNR, first, a 30% cut-off threshold from maximum was applied to the reconstructed 3D image to eliminate most background noise and artifacts. Images were then segmented in 3D space via a stack of 2D masks created by active contours with 100 iterations [35]. SNR was defined as the mean signal level of the segmented object over the mean signal level of the background.

#### 2.5.3. Methods for Volume and Iron Evaluation in The Single-Tube Phantom Series

The total phantom volume of the single-tube phantoms visualized in the images and their iron content were calculated using MATLAB (Mathworks, Natick, MA, USA). In detail, the total volume was determined as the number of voxels in the segmented area multiplied by the voxel volume and the iron amount by the integration of iron content within the segmented area. The volumes and iron contents calculated from the images were compared with the known values of the single-tube phantoms, and arithmetic means were calculated.

## 3. Results and Discussion

### 3.1. MCP 3 Synthesis

MCP 3 were synthesized according to our synthesis described previously [26]. In brief, we used a modified technique of alkaline coprecipitation of ferrous chloride with subsequent oxidation and annealing. The resulting MCP were coated with CMD for electrosteric stabilization of the MNP. We chose the coprecipitation method for developing these MNP because it is efficient and relatively mild in terms of reaction conditions. Contrast agents based on iron oxide nanoparticles and formerly used for clinical MRI of the liver (Resovist^®^ [36,37] and Endorem^®^ [37]) were developed using the coprecipitation method. 

For the present study, we optimized our initial synthesis method in terms of MPS/MPI performance by different modifications such as enlarging the approach with an upscaling factor of three and an altered magnetic separation procedure. These modifications led to better control over the magnetic core diameters and their size distribution (for details, please see supplement text S1). 

### 3.2. MNP Characterization

**Figure 1:**
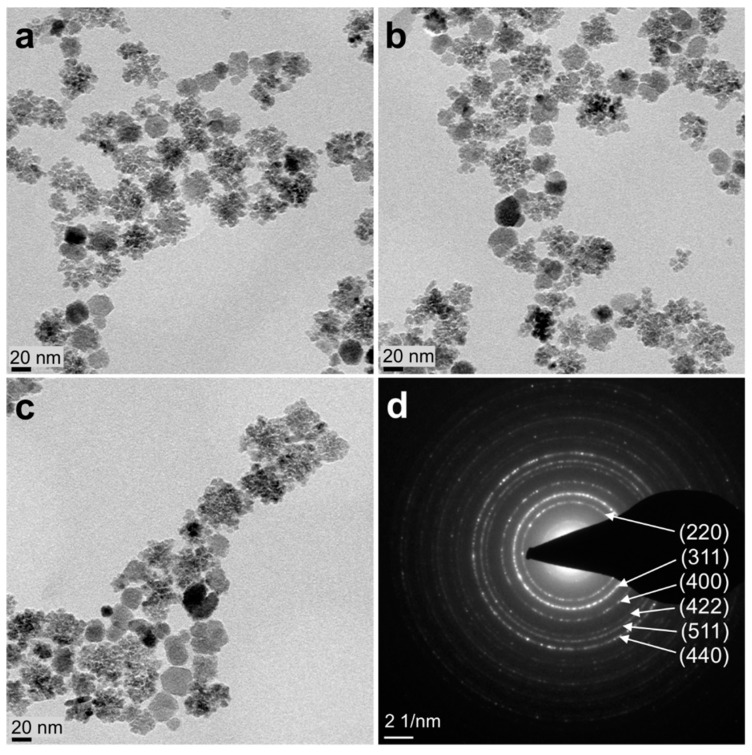
(**a–c**) TEM images of multicore particles (MCP 3) (scale bar: 20 nm) and (**d**) corresponding selected area electron diffraction (SAED) pattern (scale bar: 2 nm^−1^) For magnified TEM images of MCP 3, please see supplement Figures S2–S4.

TEM for assessment of the core structure of MCP 3 revealed that cores of MCP 3 were not uniform (Figure 1a–c), consisting of a predominantly (about 60%) clustered structure and a smaller share (about 40%) of other MNP with an unknown structure (see supplement Figure S2) [26]. The selected area electron diffraction (SAED) pattern indicates that the MNP consist of magnetite and/or maghemite (Figure 1d). The photometrically measured Fe^2+^ content was 3.9%, which strongly suggests a high maghemite content of MCP 3. For evidence of the exact iron oxide composition, for example, x-ray diffraction or Mössbauer spectroscopy investigations would be necessary [38,39].

**Figure 2:**
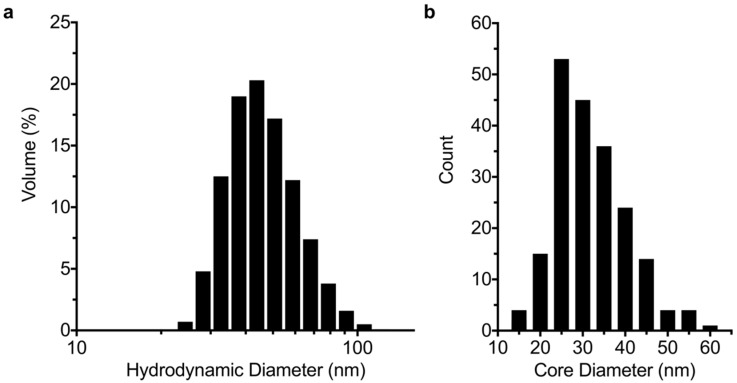
(**a**) Hydrodynamic and (**b**) TEM core diameter distributions of MCP 3. The hydrodynamic size distribution is given by volume. The TEM size distribution is based on the measurement of 200 multicore particles of MCP 3. The y-axis of the histogram in b gives the number of magnetic nanoparticles (MNP).

The hydrodynamic size of the MNP was measured by DLS. This method demonstrated the absence of aggregates, which is a very important parameter for dispersion stability and in vivo use [5]. Furthermore, the MNP dispersions can be stored for more than a year and remain stable. Figure 2 shows the hydrodynamic volume-weighted size distribution of the MNP as measured by DLS (Figure 2a) and the number-weighted size distribution determined by TEM (Figure 2b). The distribution parameters are compiled in Table 1. MNP had a mean core diameter of 32 ± 8 nm in TEM. The hydrodynamic diameter range (by volume) of the tracers measured by DLS was 24.4–122.4 nm, with a z-average of 54 nm and a polydispersity index (PDI) of 0.08. 

**Figure 3:**
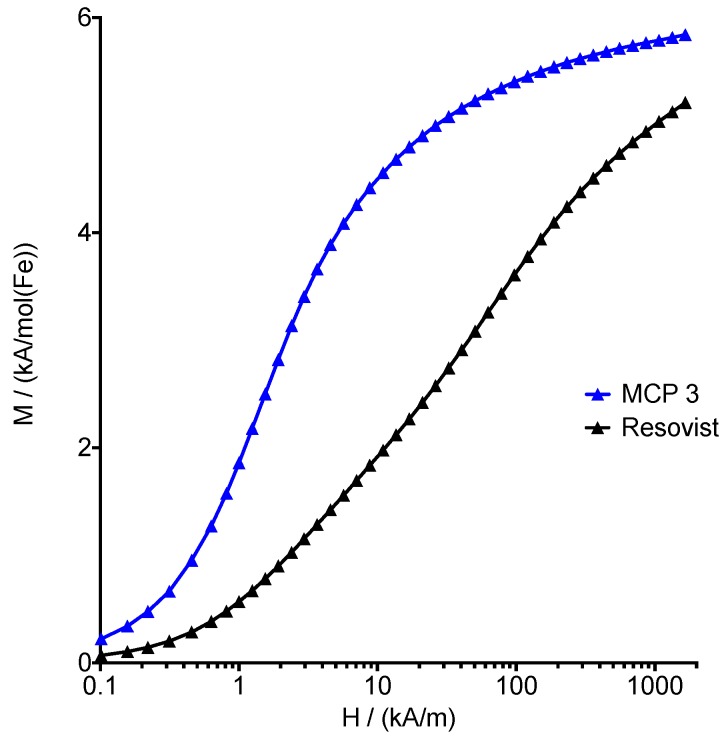
Molar magnetization *M* as a function of applied external field *H* measured for MCP 3 and Resovist^®^ at 295 K.

The M(H) curve derived from the measurements revealed a saturation magnetization *M*_S_ for MCP 3 of 5.8 ± 0.3 Am^2^/mol(Fe) (104 ± 4 Am^2^/kg(Fe)) (Figure 3), which is close to *M_S_* bulk values of 6.2 Am^2^/mol(Fe) (111 Am^2^/kg(Fe)) for maghemite reported in the literature [26]. This fits well with the low photometrically measured Fe^2+^ content of 3.9% and the presumed high maghemite content of the magnetic cores. It should be noted here that the saturation magnetization of magnetite/maghemite nanoparticles is usually below pure bulk values [40,41,42,43,44,45], which is attributable to surface effects and/or a high amount of crystallographic disorder [26]. For comparison, the *M_S_* of pure Magnetite is reported to be 7.1 Am^2^/mol(Fe) (127 Am^2^/kg(Fe)), and the Fe^2+^ content should theoretically be 33.3% [46].

**Figure 4:**
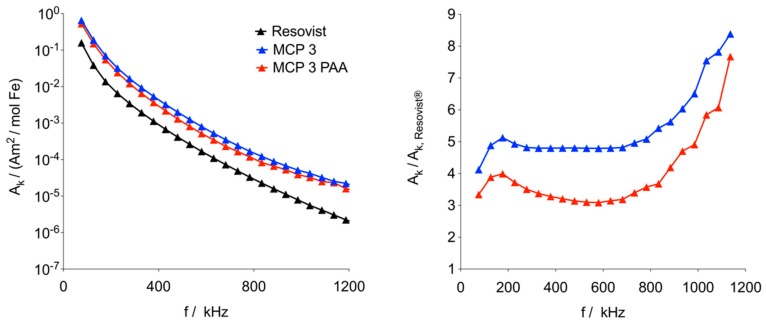
Left: magnetic particle spectroscopy (MPS) data of MCP 3 in aqueous dispersion (blue) and polyacrylamide (PAA) gel (red) in comparison with Resovist^®^ (black) at 10 mT and 25 kHz. Data are plotted as magnetic moment (normalized to iron content) versus frequency. Right: The ratio of amplitudes of MCP 3 and Resovist^®^ in aqueous dispersion (blue) and in the PAA gel matrix (red). In both cases, only odd harmonics are shown, and lines were added to guide the eye.

MPS inductively measures the nonlinear response of the MNP to an alternating magnetic field [47]. In an MPS, the measured intensities of the harmonics of the basic frequency are a measure of the potential properties in an MPI scanner. MPS can be regarded as a zero-dimensional MPI scanner without spatial resolution [47]. Therefore, MPS is a very important tool for the fast screening of different MNP probes with regard to their MPI properties in MPI tracer development. MPS measurements showed markedly stronger signal amplitudes for MCP 3 compared to Resovist^®^ as the standard of reference. The signal magnitude of MCP 3 is amplified by a factor of approx. five in the relevant range of up to 800 kHz (Figure 4). In vivo, the largest proportion of MNP is assumed to be first opsonized and then phagocytozed by macrophages especially in the liver and spleen [48]. In this case, the MNP would subsequently be taken up into lysosomes and appear wholly or partially immobilized within the MPS measurement time of 40 µs. For the first assessment of the MPS properties of MCP 3 under these conditions, we embedded the MNP in a PAA gel (4.2%) [49]. In the relevant range, the MPS signal of this gel was slightly lower in amplitude but still three to four times higher than in the Resovist^®^ dispersion, i.e., immobilization reduced the signal amplitude by about 20% at the 3rd harmonic.

**Figure 5:**
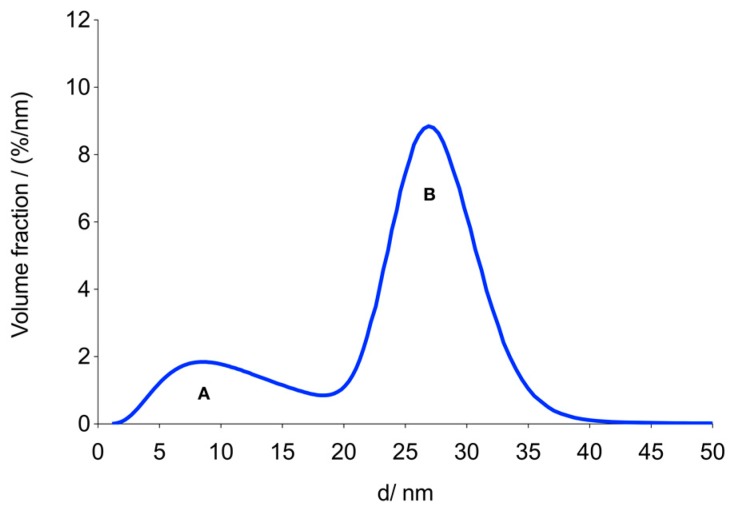
Bimodal volume distribution of MCP 3 with modes A and B calculated from the M(H) data. It is probable that only mode B contributes significantly to the MPS/MPI signal.

The signal drop of the harmonics in PAA gel may be attributable to a reduction of Brownian relaxation of MCP 3 [48,50] and/or to dipole-dipole interactions between the magnetic moments of the MNP [49,51]. The M(H) curve was analyzed using a model that describes magnetization by the superposition of non-interacting MNP of different sizes [28]. We applied a bimodal lognormal distribution of the magnetic moments, which was previously shown to be useful for the analysis of data obtained with Resovist^®^ [28]. The distribution of effective magnetic diameters was derived from the distribution of magnetic moments assuming a spherical shape of the MNP and an identical saturation magnetization for all MNP. The resulting bimodal volume-weighted distribution of the effective diameters is shown in Figure 5. The first mode, A, consists of magnetic domains with a mean diameter of about 10 nm, which do not significantly contribute to the MPS or MPI signal. We, therefore, focused on the second mode, B, whose parameters are listed in Table 2.

It is noteworthy that the mean volume diameter, *d*_v2_, of the second mode, B, is 16% smaller than the mean diameter of MCP 3 measured by TEM. The corresponding mean volumes differ by 47%. This can be explained by a particular multi-domain core structure of MCP [26]. In contrast to the structure of single-core MNP, the structure of MCP is characterized by vacancies between individual grains, making the overall physical volume greater than the magnetic one. Furthermore, the magnetization of different grains or elementary cores within the MCP may not be aligned in parallel. This also reduces the magnetic moment of the MCP. The magnetic mean volume diameter of mode B is close to the predicted optimal diameter of 25 nm for a 25 kHz excitation field frequency [29], and this part B accounts for a high fraction of 73% of the total volume. The saturation magnetization of MCP 3 is very close to that of bulk maghemite [26]. These observations could explain the high MPS/MPI signal intensity of MCP 3. 

### 3.3. MPI Phantom Measurements/Image Reconstruction

**Figure 6:**
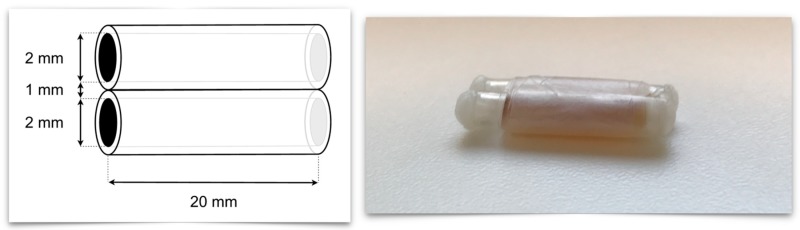
Left: Schematic drawing of a dual-tube phantom with 20 mm length and 2 mm inner diameter. Right: Photograph of a phantom with a 3 mm inner diameter filled with 10 mmol Fe/l tracer dispersion.

Sensitivity, selectivity, and spatial resolution, which can be achieved with MCP 3 as tracer were evaluated using PVC tube phantoms with 1, 2, and 3 mm inner diameters, 0.5 mm wall thickness, and 20 mm length. As a reference for comparison, phantoms filled with Resovist^®^ were used. First, we imaged single-tube phantoms to determine the SNR and spatial resolution of the two MPI tracers. Sensitivity, spatial resolution, and especially selectivity were assessed in dual-tube phantoms (Figure 6).

#### 3.3.1. Phantom Dilution Series

**Figure 7:**
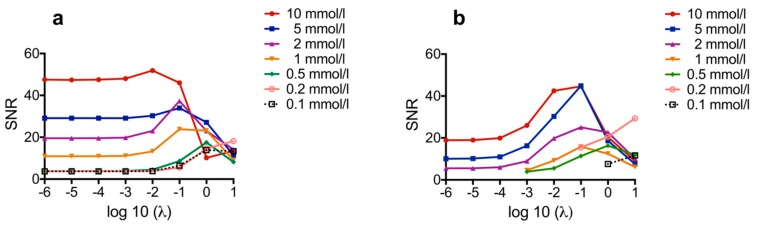
Signal-to-noise ratio (SNR) plotted over λ for MCP 3 (**a**) and Resovist^®^ (**b**). For reconstruction of the tube phantoms, the λ-values with the highest corresponding SNR for each tracer concentration were used, which were in the range of 10^−1^ to 10^−2^.

The dilution series was investigated in a range of 0.1 to 10 mmol Fe/l, which is relevant for in vivo experiments. In general, reconstruction parameters should be optimized to achieve good spatial resolution while minimizing visual noise. Image reconstruction in MPI is an ill-posed inverse problem and hence regularization is required. Regularization parameter should be optimized to achieve a good balance between image resolution and SNR [52]. A higher regularization factor reduces artifacts while at the same time leading to a loss of object geometry, and it has been shown that variations in regularization can contribute to over- or underestimation of the size of a depicted object [53]. Accordingly, standardization of results is important for comparison. For optimal reconstruction of the tube phantoms, the regularization factor λ had to be identified that provides the best SNR for the respective tracer concentration. For this purpose, the MPI measurement data of the single-tube phantoms measured with their long axis in x-direction (x orientation) were reconstructed with different regularization factors λ for each measured tracer concentration, and then SNR was plotted over λ to identify the best λ for reconstruction with the highest SNR [16] (Figure 7).

**Figure 8:**
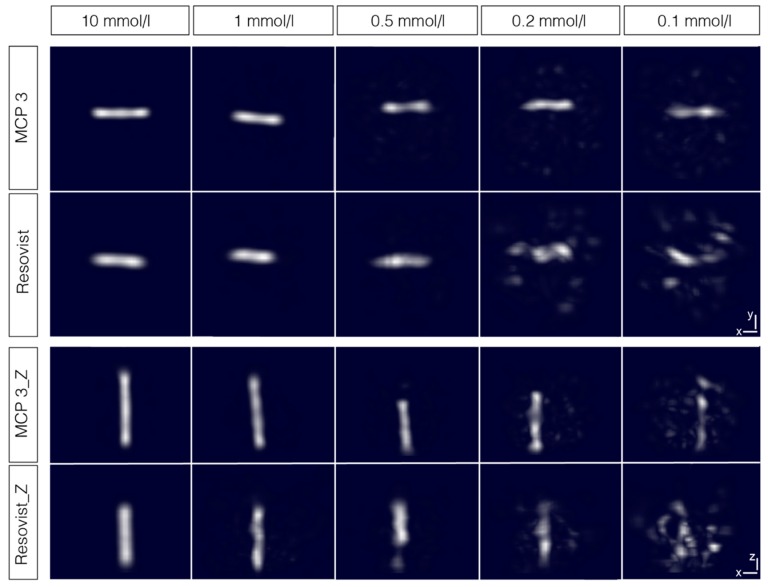
Reconstructed images of the phantom dilution series. PVC tubes with an inner diameter of 3 mm and filled with MCP 3 and Resovist^®^ at concentrations from 10 to 0.1 mM Fe. The tubes were measured in two orientations to take the different gradients of the selection field into account. Images are interpolated and represented as maximum intensity projections with the threshold set at 50%. For images of phantoms filled with 2 and 5 mmol Fe/l, please see supplement Figure S7.

The results show that both tracers have the highest SNR in the range of λ = 10^−1^ to 10^−2^, especially at lower concentrations (<2 mmol Fe/l), which might be relevant for, in vivo studies, the achievable SNR of MCP 3 is much higher than that of Resovist^®^. At higher concentrations (> 2 mmol Fe/l), the SNR of MCP 3 is rather constant at λ-values below 10^−1^. Overall, the SNR-values of all measured MCP 3 concentrations are nearly constant at λ-values below 10^−3^ and the SNR is more than two times higher. Conversely, the SNR values of Resovist^®^ strongly depend on the applied λ. The SNR-values of Resovist^®^ concentrations below 2 mmol Fe/l were only determinable in the λ-range of 10 to 10^−3^ for concentrations of 1 and 0.2 mmol Fe/l and of 1 to 10 for 0.1 mmol Fe/l. In the final reconstruction of the phantom images, λ = 10^−2^ was used for higher concentrations, e.g., 10 or 5 mmol Fe/l, and λ = 10^−1^ for lower concentrations. The reconstructed interpolated images of the phantoms of the dilution series show good reproduction of the phantoms from 10 mmol Fe/l down to 2 mmol Fe/l (Figure 8, for images of phantoms filled with 2 and 5 mmol Fe/l, please see supplement Figure S7). The phantoms were measured in two orientations, with their long axis in x- and z-direction, to take the different gradients of the selection field into account. In the range from 1 to 0.1 mmol Fe/l, the changes in phantom shapes become increasingly distorted along with an increase in artifact and noise levels. In x-axis orientation the phantoms filled with MCP 3 are reasonably well displayed down to concentrations of 0.1 mmol Fe/l. Overall, visualization was better for MCP 3-filled phantoms compared with phantoms filled with Resovist^®^, which produced only noise and artifacts especially at the lowest concentration of 0.1 mmol Fe/l. As MPI is being developed as a new medical imaging modality, recovery of the shape of the target structures to be imaged is essential. 

#### 3.3.2. Determination of Volumes and Iron Amounts in the Phantom Dilution Series

**Figure 9:**
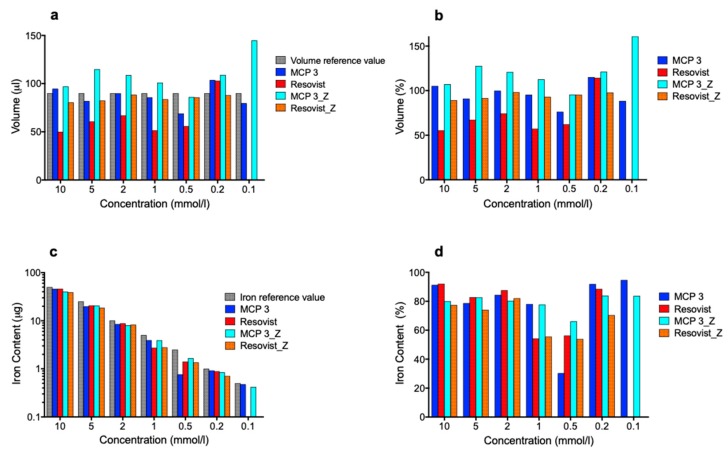
Determination of volume and iron content in the phantom dilution series: volume in absolute terms (**a**) and in relation to the reference volume (**b**), iron content in absolute terms (**c**), and in relation to the absolute iron content (**d**).

For the determination of total volumes and iron contents of the phantoms in the dilution series, the reconstructed images were segmented into two regions: the region of interest (ROI), i.e., the investigated phantom, and background. The total volume was then calculated as the sum of the voxels within the ROI, and the corresponding iron content was determined by the integration of the iron content within this region. The results are shown in Figure 9. 

The high noise and artifact levels precluded determination of the total volume and iron content of the phantoms filled with 0.1 mmol Fe/l Resovist^®^ in both x- and z-orientation. Our method allowed determination of the volumes of both tracers in the two orientations with arithmetic mean deviation of ±28% (for numerical percentages, please see supplement Tables S9 and S10). Overall, the results for MCP 3 were closer to the volume reference values of 90 µL than those for Resovist^®^. The only noticeable result for MCP 3 was the high value in z-orientation with the lowest concentration of 0.1 mmol Fe/l. This can be explained by background noise at this very low concentration and the resulting false ROI segmentation. Iron contents found for MCP 3 and Resovist^®^ were generally lower than the iron amount setpoints. This can be attributed to the threshold-based segmentation approach and partial volume effects due to the limited resolution of MPI. Another possible contributing factor is the not ideal magnetic behavior of MPI tracers with their slow response to the magnetic fields, which also limits the spatial resolution that can be achieved and is a possible reason for underestimation of volumes and iron amounts [54]. It is striking that the results for the iron content of MCP with 0.5 mmol Fe/l in x-orientation deviate from the general trend. Overall the MCP 3 values of total volume and iron content are closer to the respective setpoints than those of Resovist^®^. 

#### 3.3.3. Dual-Tube Phantoms

**Figure 10:**
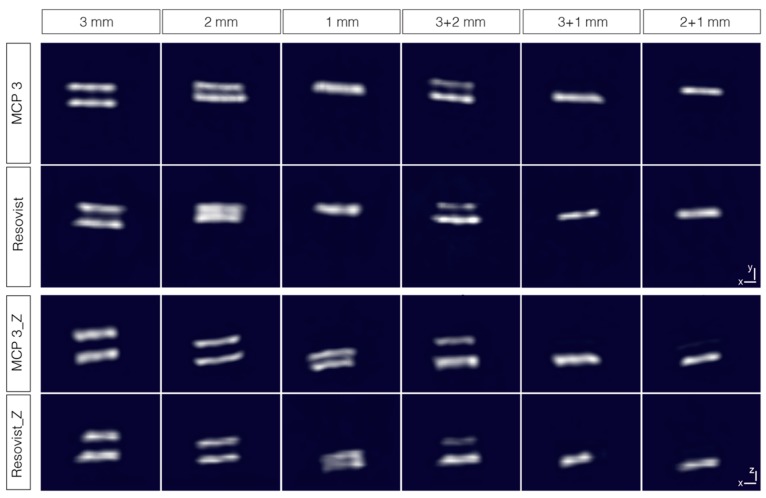
Dual-tube phantoms combining tubes of identical and different inner diameters (of 1, 2, and 3 mm) with MCP 3 and Resovist^®^ at 10 mmol Fe/l and measured in two orientations. Images are interpolated and represented as maximum intensity projections with the threshold set at 50%.

For assessing especially, the selectivity of MCP 3 with a view to potential in vivo use, all possible combinations of two tubes were assembled and filled then with 10 mmol Fe/l tracer dispersion. The resulting 12 phantoms were measured in two orientations, one parallel to the higher gradient in z direction and another parallel to a lower gradient in x direction (Figure 10). This was done considering the higher spatial resolution in the orientation of the higher gradient as a consequence of the asymmetry of the selection field.

The similar assembled dual-tube phantoms of 2 and 3 mm inner diameter are well resolved and separated from one another, except for the Resovist^®^ phantom with 2 mm inner diameter in x-y-orientation, where the two tubes are poorly separated. The phantoms with 1 mm inner diameter are indistinguishable and appear as a single object. 

The dual-tube phantoms with combinations of different inner diameters with 2 and 3 mm are well resolved and separated from one another. In the cases of phantoms with combinations of 3 mm and 1 mm as well as 2 mm and 1 mm, only the tubes with the larger diameter are clearly visible. The smaller tubes are only visible after adjustment of brightness and contrast, such as for the MCP 3 phantoms in z-orientation in both cases (please see supplement Figure S8).

A large difference in the iron amount of two objects present in the FOV results in the suppression of the object with the lower iron amount. This is known as the “shadowing effect”, which becomes more conspicuous as the ratio between the two different iron amounts increases [55]. This effect is considerably less pronounced for MCP 3 compared with Resovist^®^. The iron amount ratios for the 3 + 1 mm and 2 + 1 mm phantoms are 9:1 and 4:1, respectively.

**Figure 11:**
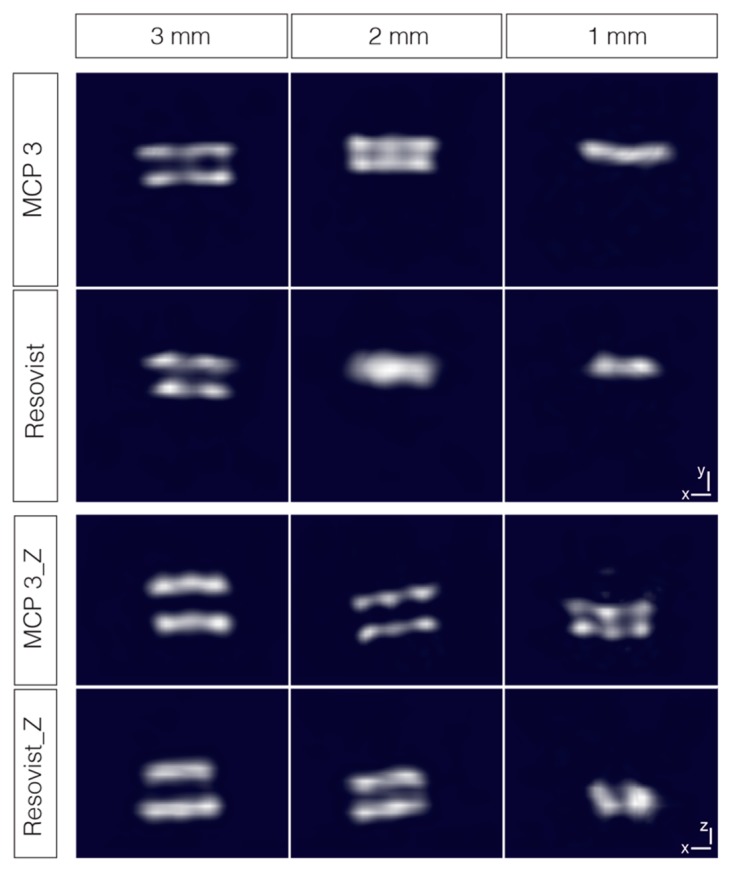
Dual-tube phantoms of 3, 2, and 1 mm inner diameter, filled with 1 mmol Fe/l MCP 3 and Resovist^®^ and measured in two orientations. Images are interpolated and represented as maximum intensity projections with the threshold set at 50%.

In addition, the MPI properties of MCP 3 were also assessed with similar combined dual-tube phantoms with 3, 2 and 1 mm inner diameter filled with 1 mmol Fe/l MCP 3 and Resovist^®^ (Figure 11). The results for the 3- and 2-mm phantoms are consistent with the results obtained for 10 mmol/l, except for the 2-mm phantoms in x-y-orientation, which appears to be the limit for differentiation of the two tubes using MCP 3. The equivalent Resovist^®^ phantom is visually one object. All 1-mm phantoms are visually one object in x-y orientation and show artifacts in x-z orientation. In the x-z-orientation of the MCP 3-filled 1-mm phantom, one can guess the existence of two tubes, but they are not clearly separated from each other. 

In summary, the dual-tube phantoms prepared with the lower concentration of 1 mmol Fe/l MCP 3 provide more selectivity, spatial resolution, and a lower artifact level compared with the corresponding phantoms prepared with Resovist^®^, and the two tubes were distinguishable down to an inner diameter of 2 mm.

## 4. Conclusions

The new MCP 3 generates an MPS signal amplitude which is about five times stronger than the signal achieved with Resovist^®^. Embedding of the MNP in PAA gel is a good simulation of the fully or partially immobilized state of MNP after uptake into the liver or spleen. Even under these conditions, the signal produced by MCP 3 is still three to four times higher compared with Resovist^®^. These excellent MPS results were also confirmed by our phantom experiments in the MPI scanner. The evaluation of the optimal regularization factor λ also indicated the higher SNR for MCP 3. These results are also consistent with the observed better resolution and better visualization of phantom geometry seen for MCP 3 versus Resovist^®^. MCP 3 also shows excellent selectivity in the dual-tube phantom experiments in both orientations parallel to the higher gradient in z direction and the lower gradient in x direction. Our results demonstrate the superiority of MCP 3 over Resovist^®^ for all investigated MPI characteristics, suggesting that MCP 3 is a promising candidate for future in vivo studies. The strong shadowing effect, observed especially for Resovist^®^, suppresses the signal of smaller iron amounts in the presence of larger ones and might be a potential challenge in prospective in vivo investigations, for instance in the imaging of adjacent blood vessels of different diameters. Compared to our previously presented initial MCP the new MCP 3 show a much higher MPS/MPI signal, which could be achieved by optimizing the synthesis regarding a better control over the resulting magnetic core diameters. The results obtained with MCP 3 also show that the coprecipitation method is well suitable to develop high-performance MPI tracers.

## Figures and Tables

**Table 1:** Compilation of characteristics of MCP 3 as determined by TEM and DLS.

Mean Core Diameter (TEM) ^*^ [nm]	d_V_ (DLS) [nm] by Volume	Z-Average (DLS) [nm]	PDI (DLS)	ζ-Potential [mV]
31.72 ± 8.4	24.4–122.4	53.94	0.08	−33.5

^*^: 200 MCP counted.

**Table 2:** Fit parameters obtained from the analysis of the M(H) data.

Sample	*β*	*d*_v2_ (nm)	*σ*_2_	*M*_s_ Am^2^/kg Fe	*μ_2_* (aAm^2^)	*β·μ_2_* (aAm^2^)	*M*_3_ Am^2^/mol(Fe)
MCP 3	0.73 ± 0.02	27 ± 0.5	0.13 ± 0.01	5.81 ± 0.25	3.9 ± 0.2	2.81 ± 0.16	0.65
*β*	Volume fraction of the second mode (B) of the assumed bimodal size distribution obtained from M(H) data						
*d*_v2_	Mean volume diameter of the second mode (B) of the assumed bimodal size distribution obtained from M(H) data						
*σ*_2_	Geometric dispersion parameter of the second mode (B) of the assumed bimodal size distribution obtained from M(H) data						
*M*_s_	Saturation magnetization of the second mode (B) of the assumed bimodal size distribution obtained from M(H) data						
*μ*_2_	Mean magnetic moment of the second mode (B) of the assumed bimodal size distribution obtained from M(H) data						
*M*_3_	Third harmonic of measured MPS data (f = 25 kHz, B = 10 mT)						

## Supplementary Materials

The following are available online at https://www.mdpi.com/2079-4991/9/10/1466/s1, Text S1: Synthesis of MCP 3, Figure S2: Magnified TEM image of MCP 3 (1/3), Figure S3: Magnified TEM image of MCP 3 (2/3), Figure S4: Magnified TEM image of MCP 3 (3/3), Figure S5: DLS data of MCP 3 (volume and intensity data, mean of 6 measurements), Text S6: System function parameters, Figure S7: Dilution series concentrations 2 and 5 mmol Fe/l (Images are interpolated and represented as maximum intensity projection with threshold set at 50%.), Figure S8: Dual-tube phantoms filled with 10 mmol Fe/l MCP 3 and Resovist^®^ processed with extreme brightness and contrast values, Table S9: Percent values of the dilution series data analysis (volume measurement), Table S10: Percent values of the dilution series data analysis (iron content).
