# Supplementary material for: MPI Phantom Study with A High-Performing Multicore Tracer Made by Coprecipitation
Source: Nanomaterials (Basel). 2019 Oct 16;9(10):1466. doi: 10.3390/nano9101466 (PMC6835925; doi:10.3390/nano9101466)
Supplement: Supplementary file 1 [file nanomaterials-09-01466-s001.pdf]

Supplementary Materials

MPI Phantom Study with A High-Performing Multicore Tracer Made by Coprecipitation

Harald Kratz [1,*], Azadeh Mohtashamdolatshahi [1], Dietmar Eberbeck [2], Olaf Kosch [2], Ralf Hauptmann [1], Frank Wiekhorst [2], Matthias Taupitz [1], Bernd Hamm [1] and Jörg Schnorr [1].

[1] Charité-Universitätsmedizin Berlin, corporate member of Freie Universität Berlin, Humboldt-Universität zu Berlin, and Berlin Institute of Health, Department of Radiology, D-10117 Berlin, Germany; Azadeh.Mohtashamdolatshahi@charite.de (A.M.); Ralf.Hauptmann@charite.de (R.H.); Matthias.Taupitz@charite.de (M.T); Bernd.Hamm@charite.de (B.H); Joerg.Schnorr@charite.de (J.S.)

[2] Physikalisch-Technische Bundesanstalt, D-10587 Berlin, Germany; Dietmar.Eberbeck@ptb.de (D.E.); Olaf.Kosch@ptb.de (O.K.); Frank.Wiekhorst@ptb.de (F.W.)

* Correspondence: Harald.Kratz@charite.de, Tel.: +49-30-450-527180

Text S1: Synthesis of MCP 3

11.88 g (59.76 mmol) Fe(II)chloride tetrahydrate was dissolved in 600 mL of deionized water under an air atmosphere (23 wt% oxygen) and stirred for 5 min at room temperature. Thereafter, 132 mL of potassium hydroxide (1 N) was added. After stirring for 10 min, 6 mL of hydrogen peroxide (5% in water) was added and the solution was stirred for 10 min subsequently, magnetic separation was realized for 15 min, the supernatant was decanted and rejected. The sediment was mixed up with 600 mL of deionized water and placed on a magnet for another 15 min. After that 24.08 g of carboxymethyl dextran sodium salt (CMD-Na) was added under stirring and stirred for 10 min at room temperature. The mixture (about 900 mL) was heated at 90 °C for 450 min. Thereafter magnetic separation was performed for 20 min, the supernatant was decanted and the sediment suspended in 500 mL of water and subjected to another magnetic separation for 20 min. This process (magnetic separation, removing of the supernatant and following resuspension with water) was repeated until the resulting supernatant is turbid. The first supernatant was removed but the following supernatants were collected, combined and washed with water via ultrafiltration using a Vivaflow 200 filter (100 kDa RC) until the filtrate has a conductivity value of less than 10 µS and concentrated to about 200 mL. The dispersion was placed on a magnet for 25 min and about 180 mL was removed by pipette and discarded, the sediment was mixed up with 180 mL deionized water, the pH of the dispersion was adjusted to a value of 11.4 by adding of 1 N potassium hydroxide solution and placed on a magnet for 25 min. Thereafter about 180 mL of the supernatant was removed and placed on a magnet for 72 h, the supernatant (155 mL) was removed and passed through 0,2 µm (Cellulose mixed ester (CME)) syringe filter (sterile filtration).

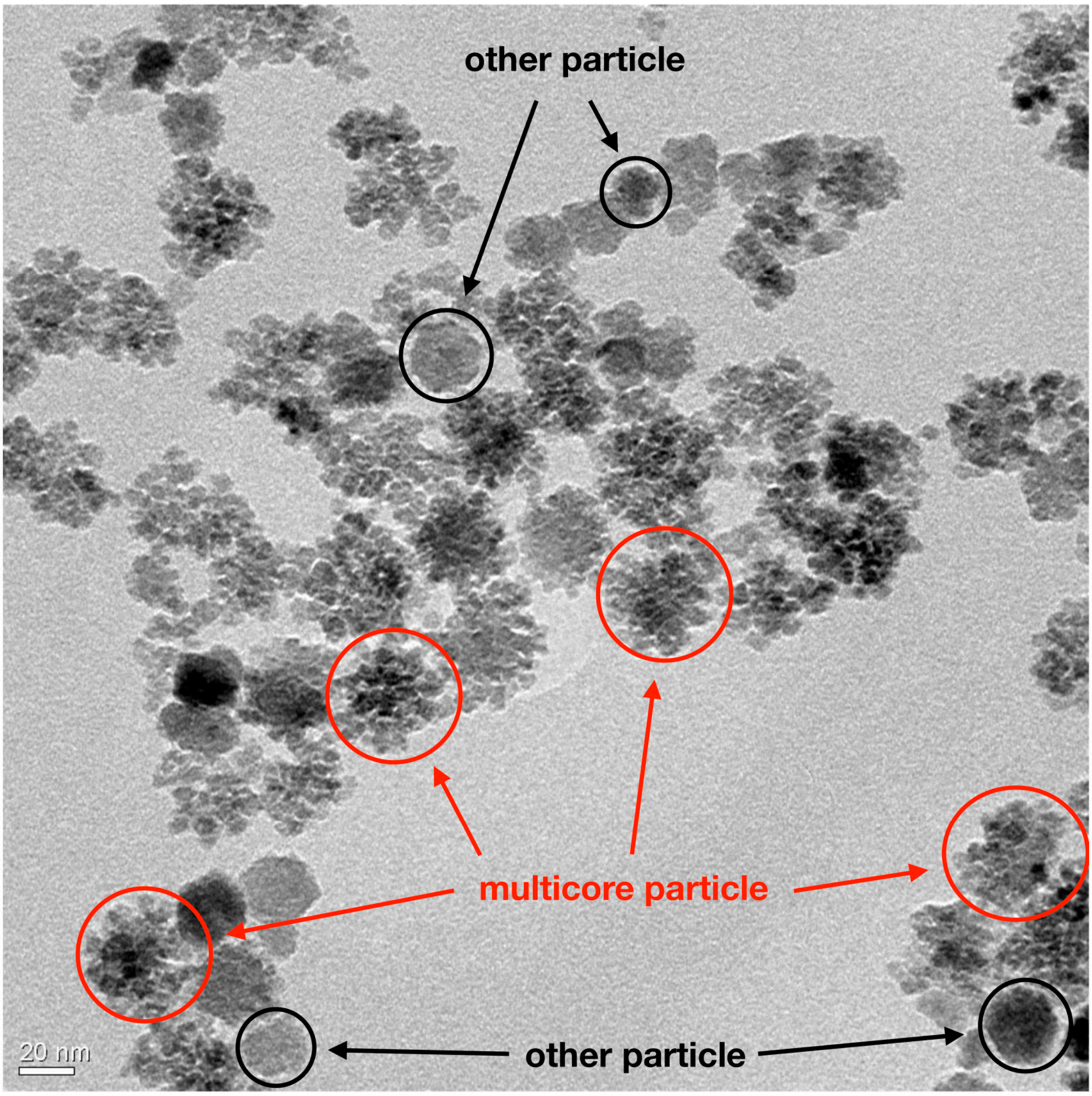

Figure S2: Magnified TEM image of MCP 3 (1/3)

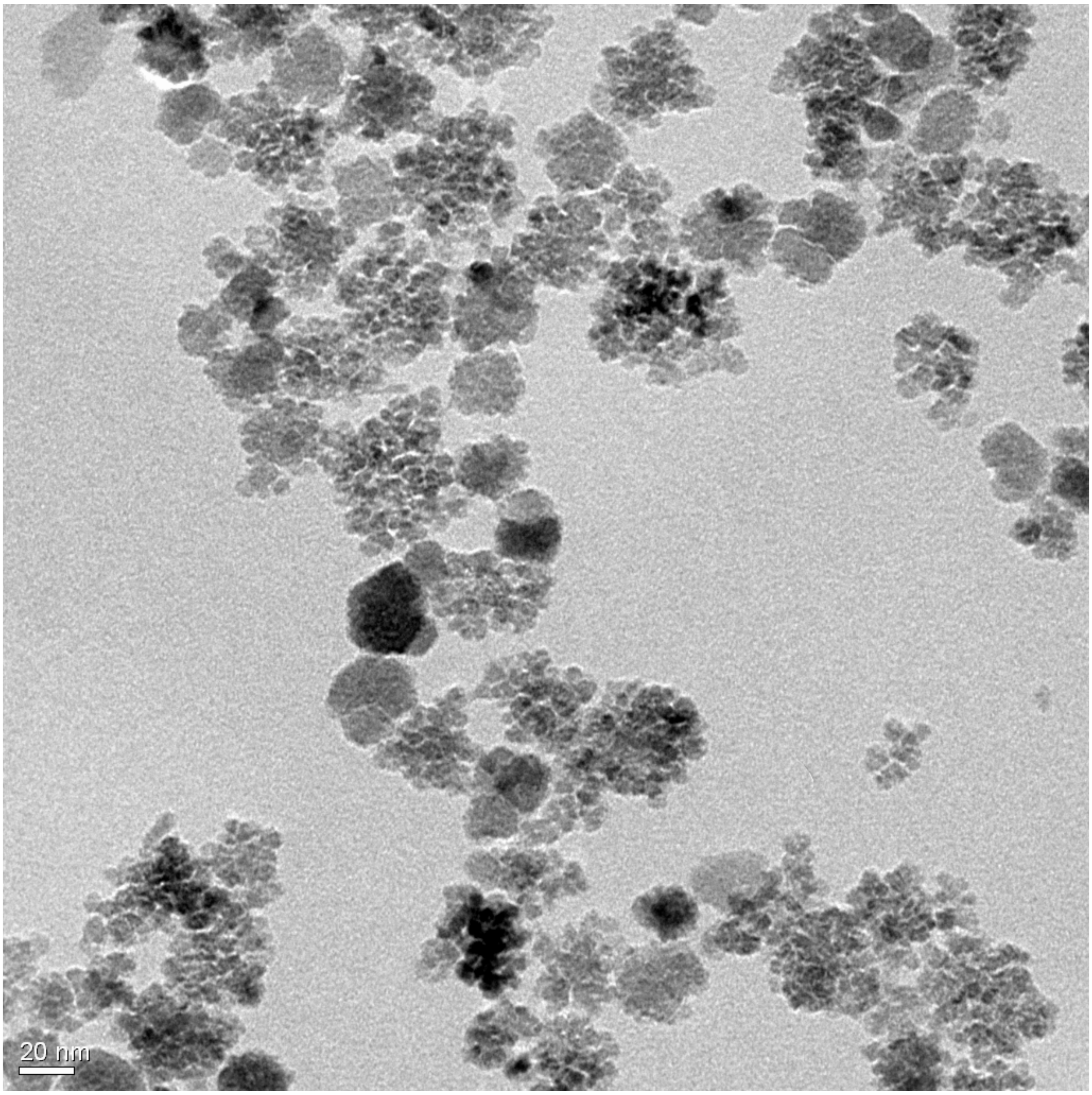

Figure S3: Magnified TEM image of MCP 3 (2/3)

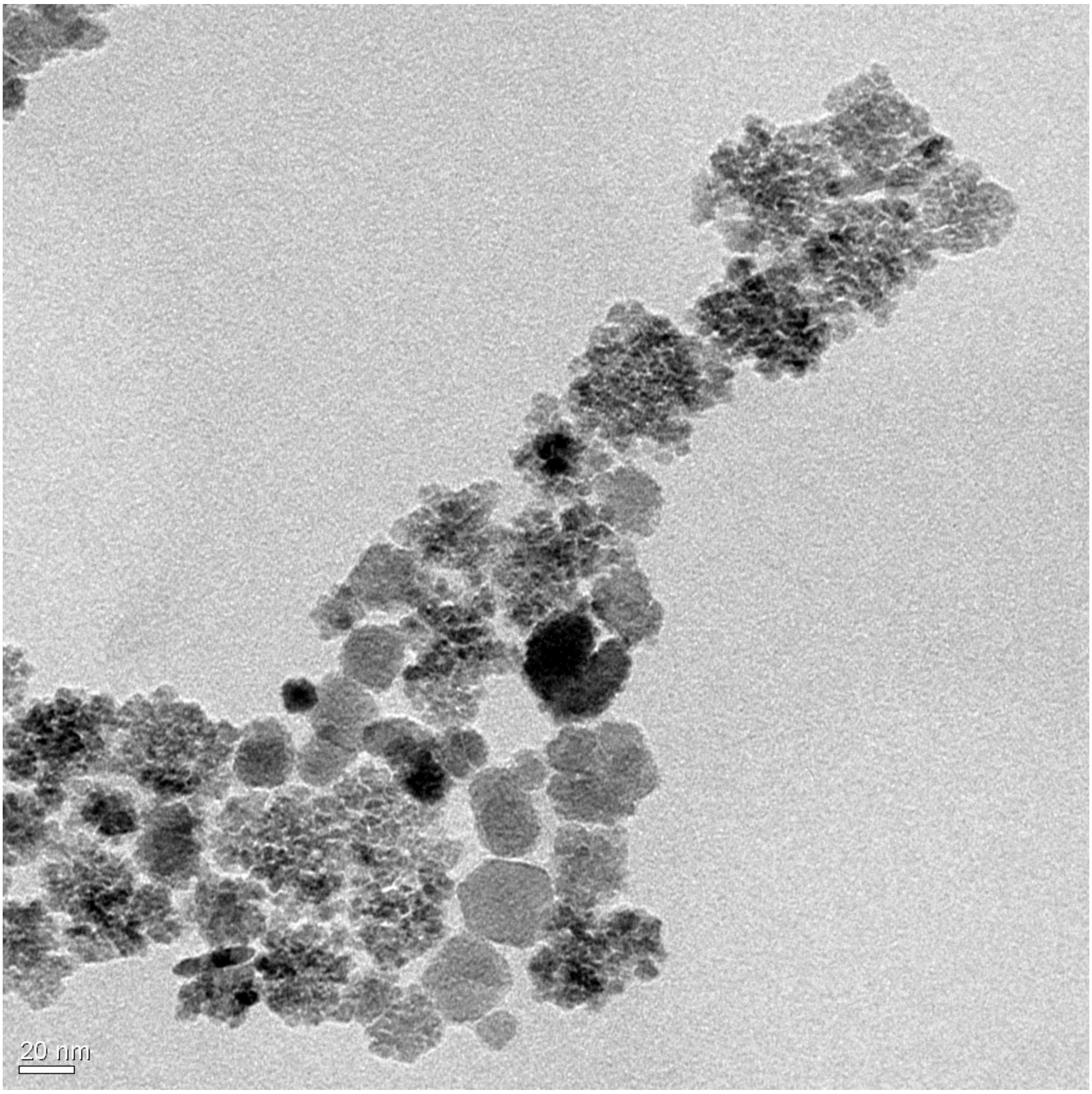

Figure S4: Magnified TEM image of MCP 3 (3/3)

Statistics Graph (1 measurements)

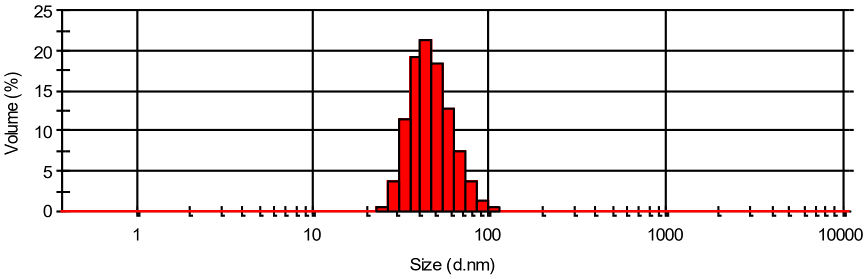

Size	Mean		Size	Mean
d.nm	Volume %		d.nm	Volume %
0,4	0		78,82	3,6
0,4632	0		91,28	1,3
0,5365	0		105,7	0,3
0,6213	0		122,4	0
0,7195	0		141,8	0
0,8332	0		164,2	0
0,9649	0		190,1	0
1,117	0		220,2	0
1,294	0		255	0
1,499	0		295,3	0
1,736	0		342	0
2,01	0		396,1	0
2,328	0		458,7	0
2,696	0		531,2	0
3,122	0		615,1	0
3,615	0		712,4	0
4,187	0		825	0
4,849	0		955,4	0
5,615	0		1106	0
6,503	0		1281	0
7,531	0		1484	0
8,721	0		1718	0
10,1	0		1990	0
11,7	0		2305	0
13,54	0		2669	0
15,69	0		3091	0
18,17	0		3580	0
21,04	0		4145	0
24,36	0,3		4801	0
28,21	3,6		5560	0
32,67	11,6		6439	0
37,84	19,2		7456	0
43,82	21,4		8635	0
50,75	18,3		1,00E+004	0
58,77	12,8			
68,06	7,5			

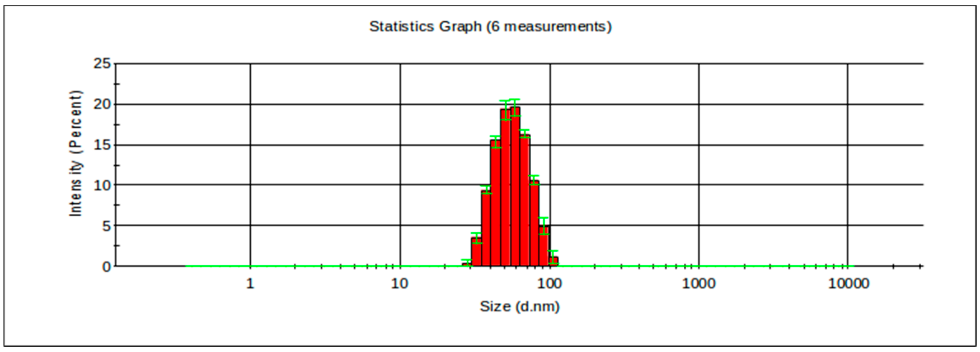

Size	Mean	Std Dev		Size	Mean	Std Dev
d.nm	Intensity Percent	Intensity Percent		d.nm	Intensity Percent	Intensity Percent
0,4	0	0		78,82	10,6	0,5
0,4632	0	0		91,28	4,9	0,9
0,5365	0	0		105,7	1,1	0,6
0,6213	0	0		122,4	0	0
0,7195	0	0		141,8	0	0
0,8332	0	0		164,2	0	0
0,9649	0	0		190,1	0	0
1,117	0	0		220,2	0	0
1,294	0	0		255	0	0
1,499	0	0		295,3	0	0
1,736	0	0		342	0	0
2,01	0	0		396,1	0	0
2,328	0	0		458,7	0	0
2,696	0	0		531,2	0	0
3,122	0	0		615,1	0	0
3,615	0	0		712,4	0	0
4,187	0	0		825	0	0
4,849	0	0		955,4	0	0
5,615	0	0		1106	0	0
6,503	0	0		1281	0	0
7,531	0	0		1484	0	0
8,721	0	0		1718	0	0
10,1	0	0		1990	0	0
11,7	0	0		2305	0	0
13,54	0	0		2669	0	0
15,69	0	0		3091	0	0
18,17	0	0		3580	0	0
21,04	0	0		4145	0	0
24,36	0	0		4801	0	0
28,21	0,2	0,3		5560	0	0
32,67	3,5	0,5		6439	0	0
37,84	9,3	0,3		7456	0	0
43,82	15,4	0,6		8635	0	0
50,75	19,3	1		1,00E+04	0	0
58,77	19,6	0,9				
68,06	16,2	0,3				

Figure S5: DLS data of MCP 3 (volume and intensity data, mean of 6 measurements)

Text S6: System function parameters

Volume of tracer: 4 μL
Sample Size: 2 x 2 x 1 mm³
Concentration of tracer: 0.072 mol Fe/l (MCP 3) and 0.1 mol Fe/l (Resovist®)
Geometrical Size: 26.4 × 26.4 × 13.2 mm³
Grid: 33 × 33 × 33
Voxel size: 0.8 × 0.8 × 0.4 mm³
Drive field amplitude: 12 mT
Excitation filed frequency: about 25kHz

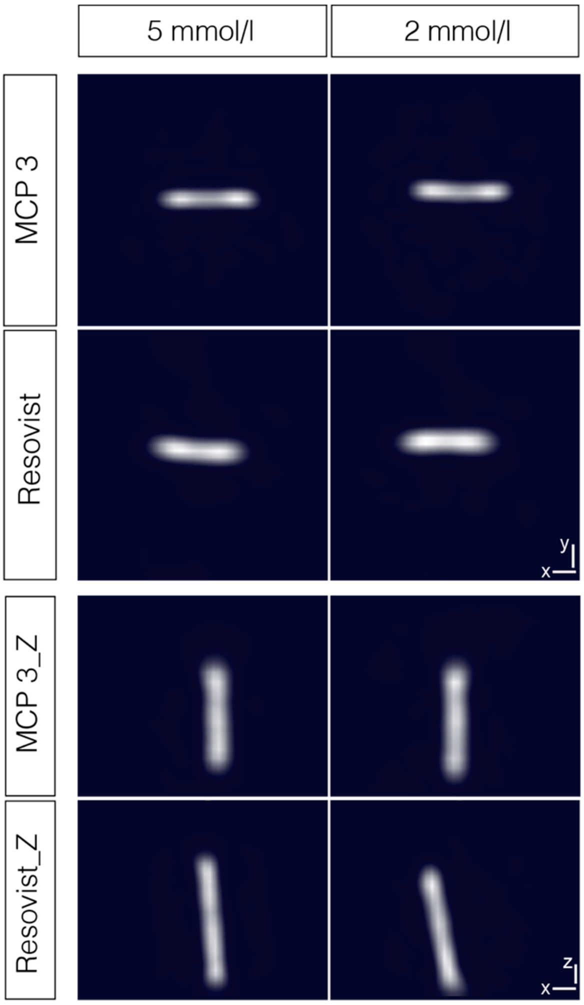

Figure S7: Dilution series concentrations 2 and 5 mmol/l Fe (Images are interpolated and represented as maximum intensity projection with threshold set at 50%.)

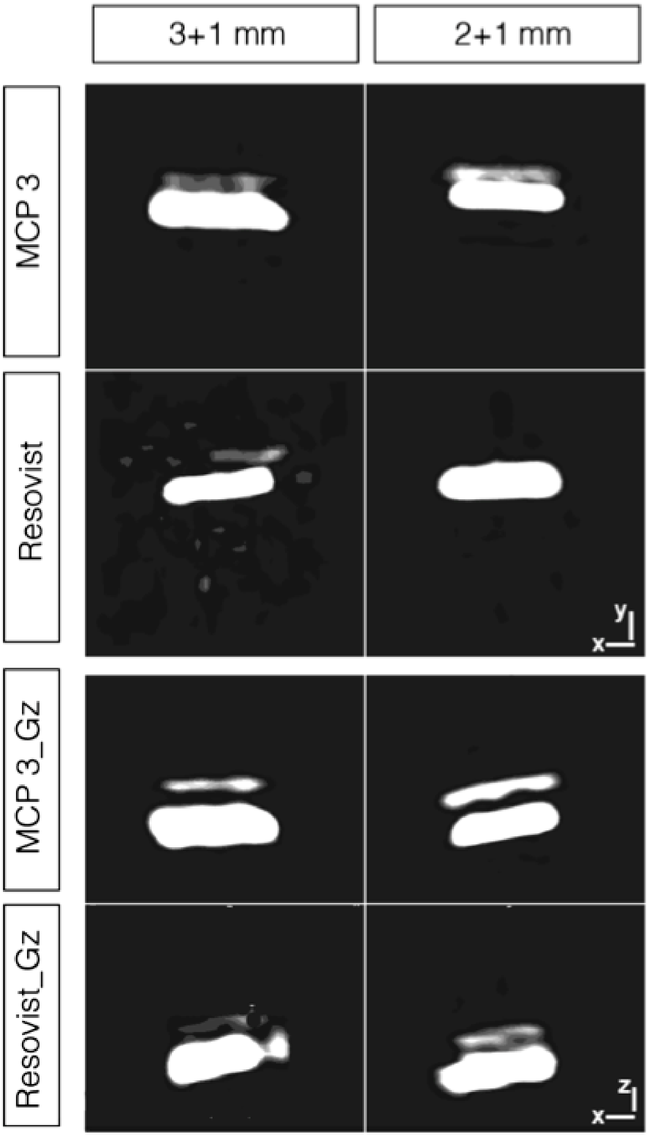

Figure S8: Dual-tube phantoms filled with 10 mmol Fe/l MCP 3 and Resovist® processed with extreme brightness and contrast values

Table S9: Percent values of the dilution series data analysis (volume measurement)

Volume measurement

(volume: 90 µl)

iron concentration	MCP with 0.1 mmol Fe/l: MCP 3 value in %	difference %	Resovist without 0.1 mmol Fe/l: Resovist value in %	difference %
10 mmol Fe/l	105,2	5,2	55,4	-44,6
5 mmol Fe/l	91,0	-9,0	67,4	-32,6
2 mmol Fe/l	99,8	-0,2	74,4	-25,6
1 mmol Fe/l	95,3	-4,7	57,2	-42,8
0.5 mmol Fe/l	76,2	-23,8	62,1	-37,9
0.2 mmol Fe/l	115,1	15,1	114,3	14,3
0.1 mmol Fe/l	88,5	-11,5	-	-
arithmetic mean	95,9	-4,1	71,8	-28,2

(volume: 90 µl)

iron concentration	MCP_Z with 0.1 mmol Fe/l: MCP 3_Z value in %	difference %	Resovist_Z without 0.1 mmol Fe/l: Resovist_Z value in %	difference %
10 mmol Fe/l	107,2	7,2	89,3	-10,7
5 mmol Fe/l	127,7	27,7	91,6	-8,4
2 mmol Fe/l	120,9	20,9	98,2	-1,8
1 mmol Fe/l	112,6	12,6	93,0	-7,0
0.5 mmol Fe/l	95,6	-4,4	95,3	-4,7
0.2 mmol Fe/l	121,2	21,2	97,9	-2,1
0.1 mmol Fe/l	161,0	61,0	-	-
arithmetic mean	120,9	20,9	94,2	-5,8

(volume: 90 µl)

iron concentration	MCP without 0.1 mmol Fe/l: MCP 3 value in %	difference %	MCP_Z without 0.1 mmol Fe/l: MCP 3_Z value in %	difference %
10 mmol Fe/l	105,2	5,2	107,2	7,2
5 mmol Fe/l	91,0	-9,0	127,7	27,7
2 mmol Fe/l	99,8	-0,2	120,9	20,9
1 mmol Fe/l	95,3	-4,7	112,6	12,6
0.5 mmol Fe/l	76,2	-23,8	95,6	-4,4
0.2 mmol Fe/l	115,1	15,1	121,2	21,2
arithmetic mean	97,1	-2,9	114,2	14,2

Table S10: Percent values of the dilution series data analysis (iron content)

Iron content measurement

(volume: 90 µl)

	MCP with 0.1 mmol Fe/l		Resovist without 0.1 mmol Fe/l	
iron concentration	MCP 3 value in %	difference %	Resovist value in %	difference %
10 mmol Fe/l	91,2	-8,8	92,1	-7,9
5 mmol Fe/l	78,7	-21,3	82,8	-17,2
2 mmol Fe/l	84,4	-15,6	87,5	-12,5
1 mmol Fe/l	78,0	-22,0	54,3	-45,7
0.5 mmol Fe/l	30,3	-69,7	56,3	-43,7
0.2 mmol Fe/l	91,8	-8,2	88,5	-11,5
0.1 mmol Fe/l	94,6	-5,4	-	-
arithmetic mean	*78,4*	-21,6	76,9	-23,1

(volume: 90 µl)

	MCP_Z with 0.1 mmol Fe/l		Resovist_Z without 0.1 mmol Fe/l	
iron concentration	MCP 3_Z value in %	difference %	Resovist_Z value in %	difference %
10 mmol Fe/l	80,1	-19,9	77,3	-22,7
5 mmol Fe/l	82,6	-17,4	74,0	-26,0
2 mmol Fe/l	80,2	-19,8	82,0	-18,0
1 mmol Fe/l	77,7	-22,3	55,5	-44,5
0.5 mmol Fe/l	66,1	-33,9	53,9	-46,1
0.2 mmol Fe/l	83,9	-16,1	70,4	-29,6
0.1 mmol Fe/l	83,7	-16,3	-	-
arithmetic mean	79,2	-20,8	68,9	-31,1

(volume: 90 µl)

	MCP without 0.1 mmol Fe/l		MCP_Z without 0.1 mmol Fe/l	
iron concentration	MCP 3 value in %	difference %	MCP 3_Z value in %	difference %
10 mmol Fe/l	91,2	-8,8	80,1	-19,9
5 mmol Fe/l	78,7	-21,3	82,6	-17,4
2 mmol Fe/l	84,4	-15,6	80,2	-19,8
1 mmol Fe/l	78,0	-22,0	77,7	-22,3
0.5 mmol Fe/l	30,3	-69,7	66,1	-33,9
0.2 mmol Fe/l	91,8	-8,2	83,9	-16,1
arithmetic mean	*75,7*	-24,3	78,4	-21,6
